# Penehyclidine in prevention of postoperative nausea and vomiting: a systematic review and meta-analysis of randomized controlled trials

**DOI:** 10.3389/fmed.2025.1676087

**Published:** 2025-09-30

**Authors:** Jia-Chao Lu, Yanjun Chen, Lan Lai, Qi-hong Shen

**Affiliations:** Department of Anesthesiology, Affiliated Hospital of Jiaxing University, Jiaxing, China

**Keywords:** penehyclidine, nausea and vomiting, meta-analysis, PONV, POV

## Abstract

**Background:**

Postoperative nausea and vomiting (PONV) are prevalent complications following general anesthesia. The effectiveness of penehyclidine (PHC) in reducing PONV is still debated. To address this issue, we conducted this systematic review and meta-analysis to assess both the effectiveness and safety of PHC in preventing PONV after general anesthesia.

**Methods:**

To gather relevant studies on PHC use for preventing PONV, six electronic databases (PubMed, Embase, Cochrane Library, Web of Science, China National Knowledge Infrastructure, and Wanfang Database) and trial registries were searched. Placebo-controlled trials that explored the effect of PHC on PONV in patients undergoing general anesthesia were included. The primary outcome was the incidence of PONV. Adverse events were evaluated to explore the safety of PHC. This meta-analysis was carried out using Review Manager 5.3. Risk of bias for included studies was assessed using the Cochrane risk of bias tool 2.0. Quality of evidence was assessed using Grading of Recommendations Assessment, Development, and Evaluation. Heterogeneity was explored by subgroup analyses. Publication bias was evaluated by funnel plot analysis. Additionally, trial sequential analysis was used to reduce the risk of type I error.

**Results:**

This analysis included ten randomized controlled trials with 1,427 participants. The PHC group showed a significantly lower incidence of PONV compared to the control group (risk ratio = 0.48, 95% confidence interval [0.36, 0.65]; *p* < 0.05, *I*^2^ = 68%). A reduction in postoperative nausea, vomiting, and the need for rescue antiemetic therapy was also associated with PHC.

**Conclusion:**

Our research suggests that PHC might be a new option for preventing PONV after general anesthesia.

**Systematic review registration:**

https://www.crd.york.ac.uk/PROSPERO/recorddashboard, CRD42022355743.

## Introduction

Postoperative nausea and vomiting (PONV) are prevalent complications following general anesthesia, with incidence rates ranging from 30 to 80%, depending on the type of surgery and patient population (1, 2). PONV causes significant patient discomfort and is linked to various adverse postoperative events, including reflux aspiration, electrolyte imbalance, esophageal injury, and wound dehiscence. In the Fourth Consensus Guidelines for the Management of PONV, volatile anesthetics, nitrous oxide, and postoperative opioids are identified as anesthetic risk factors (3).

The mechanism underlying PONV is multifaceted, involving various pathways and receptors, including cholinergic, dopaminergic, histaminergic, and serotonergic receptors (4, 5). Recent research has highlighted the central cholinergic system’s role in PONV, particularly the muscarinic 3 (M3) muscarinic acetylcholine receptor (6, 7). Current strategies for PONV prevention in high-risk patients are multimodal and often involve a combination of approaches targeting different pathways (3). Pharmacological prophylaxis remains the cornerstone and includes several classes of antiemetics: 5-HT_3_ (5-HT_3_) receptor antagonist (8), neurokinin-1 (NK-1) receptor antagonists (9), dopamine antagonists (10), corticosteroids (11), and anticholinergics (12). Non-pharmacological interventions, such as acupuncture (13) and ginger (14), have also been explored with varying levels of evidence supporting their use, often as adjuncts. Despite this array of options, the search for effective, well-tolerated, and cost-efficient preventive agents continues, especially those targeting specific mechanisms like the cholinergic pathway implicated in early PONV triggered by volatile anesthetics.

Penehyclidine (PHC) is a synthetic, long-acting anticholinergic agent developed in China. Pharmacodynamically, PHC acts as a competitive antagonist at both muscarinic and nicotinic acetylcholine receptors, but demonstrates high selectivity for muscarinic receptor subtypes, with the greatest affinity for M1 and M3 receptors, followed by M2, and significantly less for M4 and M5 subtypes (15). Its anti-nicotinic effect contributes to antagonism at neuromuscular junctions. Importantly, PHC readily crosses the blood–brain barrier, exerting significant central anticholinergic effects, which is highly relevant for targeting central pathways involved in PONV (16). Preliminary clinical studies have indeed suggested a beneficial effect of PHC in reducing PONV incidence (17, 18). However, this finding remains contentious. According to Ding et al., PHC did not significantly reduce the incidence or severity of PONV in laparoscopic bariatric surgery patients (19). Although PHC is currently approved in China for indications such as organophosphate poisoning and obstructive airway diseases, it is not approved by the U.S. Food and Drug Administration (FDA) for the prevention of PONV. Importantly, all clinical studies investigating PHC for PONV prevention to date, including those analyzed in this meta-analysis, constitute off-label use.

After identifying relevant randomized controlled trials (RCTs), we conducted a systematic review and meta-analysis with the primary objectives of evaluating the efficacy of PHC in preventing PONV following general anesthesia. Specifically, we aimed to: (1) assess the impact of PHC on the incidence of PONV compared to placebo or standard treatment; (2) evaluate the safety profile of PHC, focusing on adverse events.

## Methods

PRISMA guidelines were followed in the conduct of our systematic review and meta-analysis. The research has been registered with the International Prospective Register of Systematic Reviews as CRD42022355743.

### Systematic literature search

The search strategy for this systematic review and meta-analysis was comprehensive and systematic. We performed a systematic literature search across international databases (PubMed, Embase, Cochrane Library, and Web of science), Chinese databases (China Network Knowledge Infrastructure and Wanfang Database), and Trial registries (clinicaltrials.gov and the WHO International Clinical Trials Registry Platform) to identify RCTs related to PHC and PONV. The search terms included combinations of keywords such as ‘penehyclidine’, ‘postoperative nausea and vomiting’, ‘PONV’, ‘general anesthesia’, ‘antiemetic’, and ‘randomized controlled trial’. These terms were used in various combinations with Boolean operators (AND/OR) to ensure the identification of all relevant studies. We applied no language restrictions, and the search was limited to studies published up to July 31, 2025. All retrieved articles were screened for relevance, and duplicate studies were removed. The detailed search strategy for each database is available in the Supplementary materials. Additionally, we reviewed the references of the final eligible studies to identify any further relevant research.

### Criteria for selection

The inclusion criteria for the studies were based on the “PICOS” framework:

(1) Participants (P): adult patients of any American Society of Anesthesiologists physical status undergoing general anesthesia. Studies in which all patients received a standardized baseline PONV prophylaxis regimen in both groups were included, provided the only systematic difference between groups was the administration of PHC or placebo;
(2) Intervention (I): trials specifying PHC dosage and timing;
(3) Comparison (C): saline;
(4) Outcome (O): trials evaluating the incidence of PONV as an outcome;
(5) Study Designs (S): RCTs.

The exclusion criteria were as follows:

(1) Patients not receiving general anesthesia;
(2) Studies lacking available outcomes;
(3) Incomplete research, such as conference abstracts or unfinished studies;
(4) Non-RCTs.

### Extraction of data and outcomes

Initially, two independent reviewers screened for duplicate records. A review of the titles and abstracts of the trials was then conducted to determine whether they met the inclusion criteria. Following that, we reviewed the full texts of the remaining studies to determine final inclusion. The data extraction process was conducted independently by two reviewers using a standardized data extraction form. The form included sections for study design, participant characteristics, and intervention details. The extraction process was performed in a blinded manner, with both reviewers working independently to minimize bias. Any disagreements between reviewers were resolved through discussion or consultation with a third reviewer. For missing data, we made every effort to contact the corresponding authors of the included studies via email to request missing data.

The primary outcome of this study was the incidence of PONV. The temporal definition for PONV was set at 24 h postoperatively. However, if a study reported the incidence of PONV solely within a different timeframe (e.g., 48 h), it was still included in the pooled analysis. Secondary outcomes included severe PONV incidence, postoperative nausea (PON), postoperative vomiting (POV), dry mouth, headache, dizziness, urinary retention, fever, the number of patients needing rescue antiemetics, and post-anesthesia care unit (PACU) length of stay. The definition of severe PONV was not consistent across the included trials. To respect the original study designs and avoid introducing bias by imposing an arbitrary uniform definition, we extracted the outcome ‘severe PONV’ as it was defined by the authors of each primary study. Common definitions included multiple episodes of vomiting within a specified timeframe (19, 20) or severe nausea measured by a numerical analog scale score exceeding a certain threshold (17, 21).

### Evaluation of the quality and the risk

We assessed the risk of bias in the included studies utilizing the Cochrane risk-of-Bias tool for RCTs 2.0 (ROB 2.0), which contained six types of bias. Each trial was categorized as having a high, some concerns, or low risk of bias. Furthermore, we employed the Grading of Recommendations Assessment, Development, and Evaluation (GRADE) system to gauge the confidence in the evidence, classifying it into one of the four levels.

### Statistical analysis

This study was conducted by using Review Manager (Version 5.3. The Nordic Cochrane Centre, The Cochrane Collaboration, 2014. Copenhagen). For dichotomous outcomes, we calculated risk ratios (RR) along with 95% confidence intervals (CIs). For continuous outcomes, we determined mean differences (MD) and their corresponding 95% CIs. Continuous data reported as medians with interquartile ranges were converted to means and standard deviations following established methods (22, 23). Statistical significance was set at a *p*-value <0.05. Heterogeneity among studies was evaluated using the I^2^ statistic, with *I*^2^ > 50% indicating substantial heterogeneity. Statistical significance was defined as a *p*-value <0.05. Heterogeneity across studies was assessed using the *I*^2^ statistic, where *I*^2^ > 50% signaled substantial heterogeneity. For studies showing low *I*^2^ values, a random-effects model was applied due to notable clinical variability. We performed pre-specified subgroup analyses to investigate whether the effect of PHC on preventing PONV varied by type of anesthesia (TIVA, total intravenous anesthesia vs. Combined, combined intravenous and inhalation anesthesia), dosage of PHC (high- dosage, > 0.5 mg vs. low- dosage, ≤ 0.5 mg), and timing of PHC administration (before induction vs. after induction). For studies that calculated the PHC dosage based on body weight, a dosage of ≤ 0.01 mg/kg was defined as the low- dosage group, while the other was defined as the high- dosage group. To assess the robustness of our finding, we conducted sensitivity analyses by excluded studies with risk of bias to explore the impact of risk of bias on the primary outcome. In addition, we conducted a leave-one-out analysis to assess the stability of the main results. We performed a funnel plot analysis to visually assess the potential for such bias in the included studies.

Trial sequential analysis (TSA) was conducted using TSA software (version 0.9.5.10 beta) to control the risk of type I error that may arise from repeated testing when accumulating data (24). We set the type I error rate at 5% and the type II error rate at 20% (i.e., 80% statistical power). The required information size (RIS) was calculated based on the incidence of PONV in the control group derived from the included studies, a relative risk reduction (RRR) of 38.5% (25). Specifically, if the cumulative Z-curve crossed the TSA monitoring boundary, it would indicate that the evidence is sufficient to draw a conclusion and further studies are unnecessary. Conversely, if the Z-curve did not cross the boundary but entered the conventional significance area, the result might be a false positive, and more studies would be needed. If the Z-curve crossed the RIS line without crossing the boundary, it would suggest that the intervention is ineffective even with sufficient information size. This approach adheres to current recommendations for trial sequential analysis.

## Results

### Search results

**Figure 1:**
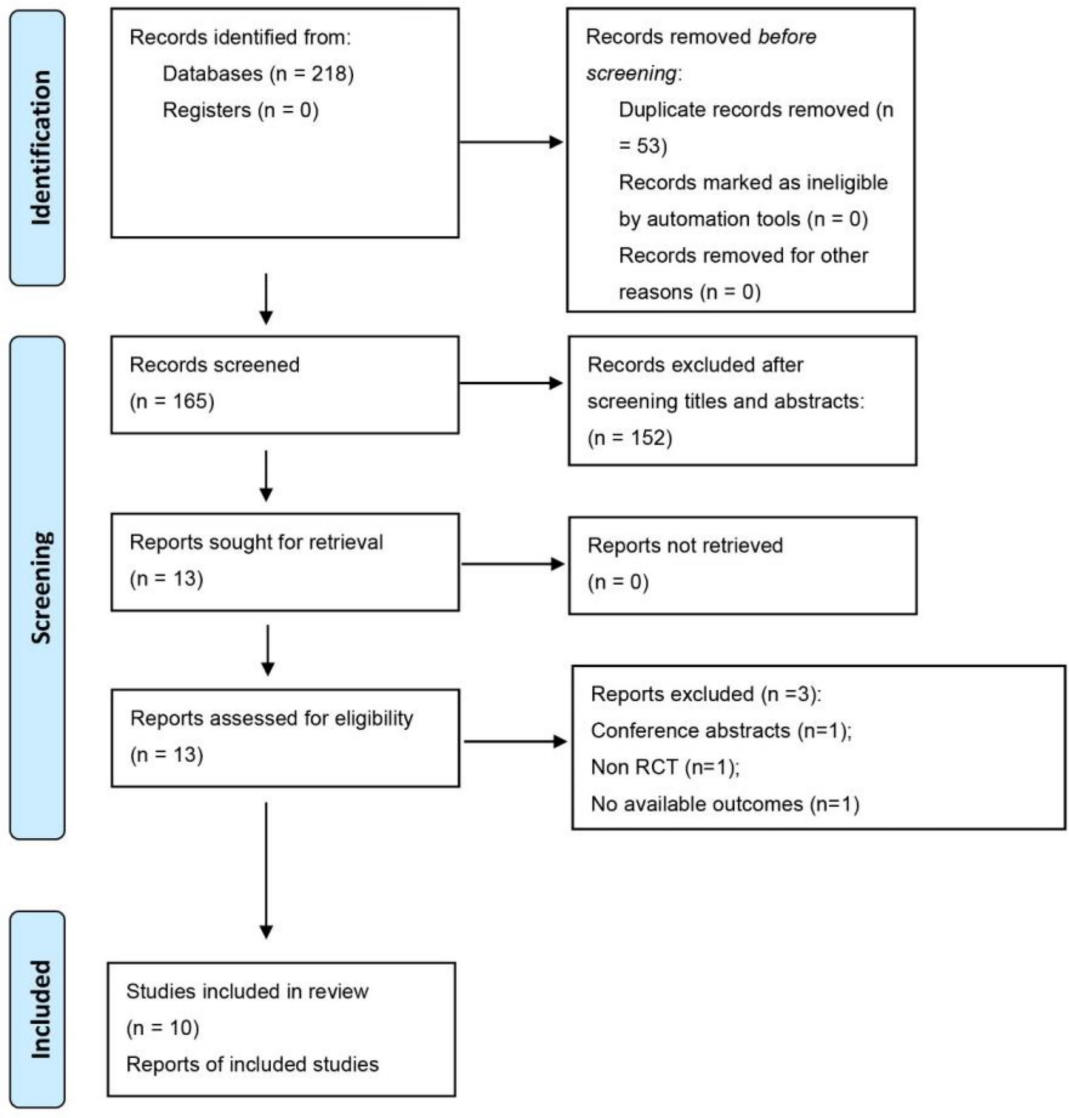
The inclusion process of the literature search.

Following our search strategy, we initially identified 218 potentially relevant studies. After removing 53 duplicate publications and excluding 152 studies based on abstract and title reviews, we assessed the full texts of the remaining 13 studies to determine their eligibility. Out of these, 3 trials were excluded for the following reasons: one was a conference abstract, one was not an RCT, and one lacked available outcome. Ultimately, 10 studies met the inclusion criteria and were included in the meta-analysis (17–21, 26–30). A detailed account of the literature screening process is illustrated in Figure 1.

### Study characteristics

The publication years between 2008 and 2024, and the sample size was ranged from 40 to 353. The type of surgeries included laparoscopic bariatric surgery, thyroidectomy, strabismus surgery, laparoscopic cholecystectomy, microvascular decompression, bimaxillary surgery and gynecological laparoscopic surgery. Three studies routinely used neostigmine to antagonize neuromuscular blocking agents after surgery (18, 20, 21). Detailed information for included studies is presented in Table 1.

**Table 1:** The details of included studies.

Study	Sample size	Type of surgery	Anesthesia induction	Anesthesia maintenance	Penehyclidine group	Control group
Ding 2023 (19)	P: 221 C: 113	Laparoscopic bariatric surgery	Dexamethasone 10 mg, midazolam 0.05 mg/kg, propofol 1.5–2.5 mg/kg, fentanyl 4–6 μg/kg, rocuronium 0.9 mg/kg or cis-atracurium 0.15 mg/kg.	Propofol 100–200 μg/kg/min, remifentanil 0.05–0.15 μg/kg/min, rocuronium 5–10 μg/kg/min or cis-atracurium 1–3 μg/ kg/min.	A single intravenous dose of 0.5 mg after anesthesia induction.	Same volume of saline.
Li 2021 (27)	P: 45 C: 45	Thyroidectomy	Midazolam 0.05 mg/kg, propofol 2 mg/kg, fentanyl 5 μg/kg, and cisatracurium 0.15 mg/kg.	Propofol 66–200 μg/kg/min, remifentanil 0.05–0.15 μg/kg/min, cisatracurium 1–3 μg/kg/min.	A single intravenous dose of 0.5 mg after anesthesia induction.	Same volume of saline.
Lu 2022 (21)	P: 50 C: 50	Total thyroidectomy	Propofol 1.5–2.5 mg/kg and fentanyl 2 μg/kg, and cisatracurium 0.15 mg/kg.	Propofol 60–200 μg/kg/min, and remifentanil 0.1–0.15 μg/kg/min.	A single intravenous dose of 0.5 mg after anesthesia induction.	Same volume of saline.
Sun 2021 (20)	P: 114 C: 104	Strabismus surgery	Propofol 1.5–2.5 mg/kg, fentanyl 5.0 μg/kg, cisatracurium 0.15 mg/kg.	Propofol 60–200 μg/kg/min, remifentanil 0.1–0.15 μg/kg/min.	A single intravenous dose of 0.01 mg/kg after anesthesia induction.	Same volume of saline.
Wang 2008 (28)	P: 20 C: 20	Microvascular decompression	Not mentioned.	Fentanyl, propofol, and isoflurane	A dose of 0.1 mg before anesthesia induction.	Same volume of saline.
Wang 2022 (22)	P1: 117 P2: 118 C: 118	Bimaxillary surgery	Sufentanil/remifentanil, propofol, and rocuronium/cis-atracurium.	Propofol and remifentanil/ sufentanil, with or without inhalational sevoflurane and/or nitrous oxide or dexmedetomidine infusion.	P1: a dose of 0.5 mg before anesthesia induction. P2: a dose of 0.25 mg before anesthesia induction; a dose of 0.25 mg was added to the intravenous analgesia pump.	Same volume of saline.
Yang 2011 (29)	P: 30 C: 30	Laparoscopic cholecystectomy	Midazolam 0.05 mg/kg, propofol 1.5 mg/kg, atracurium 0.6 mg/kg, and fentanyl 2 μg/kg.	Fentanyl and atracurium.	A dose of 1 mg before anesthesia induction.	Same volume of saline.
Zhang 2012 (18)	P: 40 C: 40	Gynecological laparoscopic surgery	Midazolam 0.08 mg/kg, fentanyl 5 lg/kg, etomidate 0.3 mg/kg, cisatracurium 0.2 mg/kg.	Propofol 3–4 lg/ml, remifentanil 3 ng/mL; muscle relaxation 0.08 mg/kg/min.	A dose of (0.01 mg/kg, maximal total dose, 1 mg) before anesthesia induction.	Same volume of saline.
Zhang 2010 (30)	P: 30 C: 30	Laparoscopic cholecystectomy	Midazolam 0.06 mg/kg, fentanyl 3 μg/kg, atracurium 0.6 mg/kg, and etomidate 0.3 mg/kg.	Propofol and remifentanil	A dose of (0.02 mg/kg) before anesthesia induction.	Same volume of saline.
Zhao 2024 (26)	P: 46 C: 46	Gynecological laparoscopic surgery	Midazolam 0.04 mg/kg, sufentanil 0.5 μg/kg, etomidate 0.3 mg/kg, rocuronium 0.8 mg/kg.	Sevoflurane 1%, remifentanil 0.1–0.3 μg/kg/min, propofol 2–5 mg/kg/h.	A bolus of 0.01 mg/kg after anesthesia induction.	Same volume of saline.

P, penehyclidine; C, control; PONV, postoperative nausea and vomiting.

### Risk of bias

**Figure 2:**
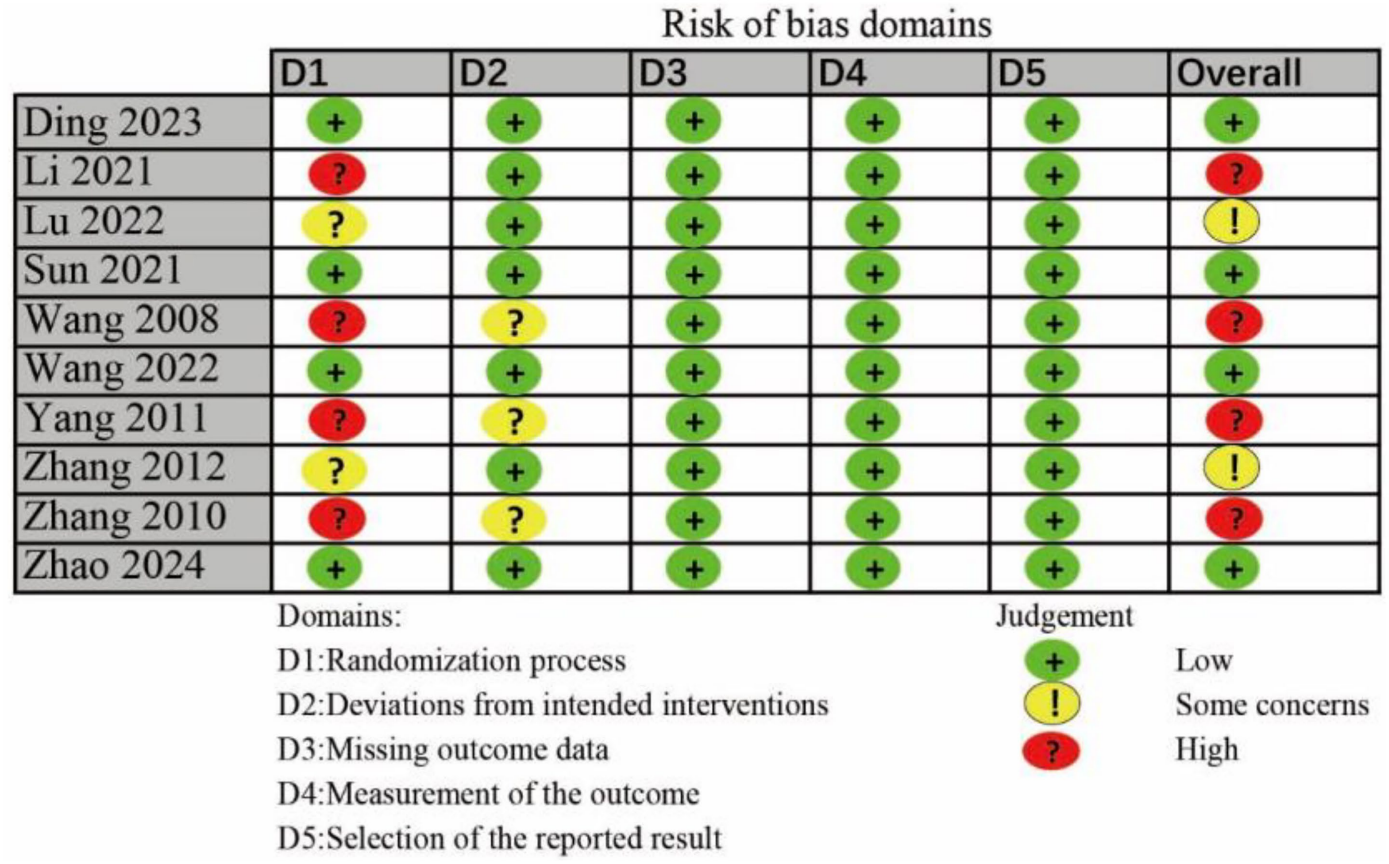
The risk bias assessment of all included studies.

Risk of bias assessment for individual studies is shown in Figure 2. Four trials had the high risk of randomization process, and two trials had some concerns risk of randomization process. Three trials had some concerns risk of deviations from intended interventions. Of the included trials, four were classified as low risk of bias, two raised some concerns, and four were considered high risk.

### Outcomes

#### Primary outcome

##### The incidence of PONV

**Figure 3:**
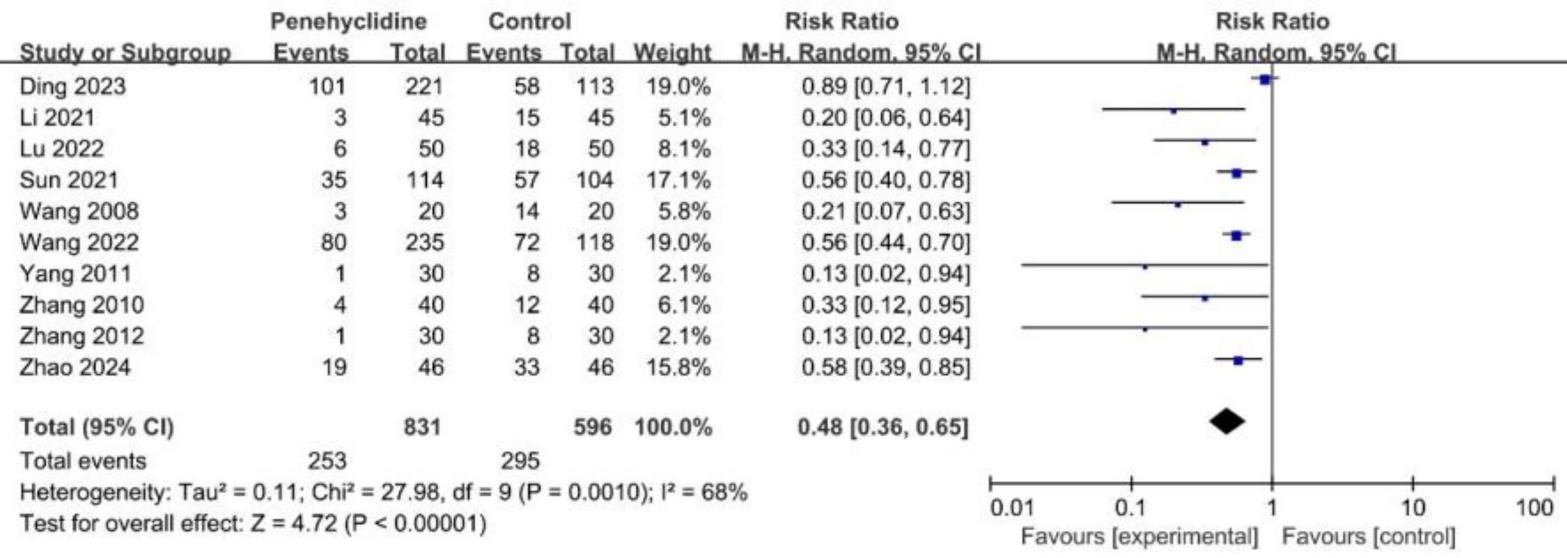
Forest plot of the incidence of PONV between penehyclidine and control groups. PONV, postoperative nausea and vomiting.

All the trials included reported on the incidence of PONV. The forest plot indicated a significant reduction in PONV rates for the PHC group (RR = 0.48, 95% CI [0.36, 0.65], *p* < 0.05, *I*^2^ = 68%, Figure 3), highlighting substantial heterogeneity among the studies. Notably, the trial by Ding et al. was identified as a major contributor to this variability. After excluding this study, we re-conducted the meta-analysis, which yielded similar results with reduced heterogeneity (Supplementary Figure 1). Further subgroup analyses also yielded results consistent with the overall finding (Supplementary Figures 2–4). Sensitivity analyses suggest that the risk of bias did not dramatically alter the overall effect estimate. Furthermore, we further evaluated the effect of PHC on preventing PONV in studies that routinely used neostigmine for neuromuscular blockade reversal after surgery, and consistent result was obtained (RR = 0.42, 95% CI [0.23, 0.77], *p* < 0.05, *I*^2^ = 41%). In addition, the results of the leave-one-out analysis confirmed that the primary outcome was stable.

The incidence of PONV was 49.5% in the control group, and 30.4% in the PHC group, the absolute risk reduction (ARR) was 19.1%. The number needed to treat (NNT) to prevent one case of PONV was 5.2.

#### Secondary outcomes

##### PON occurrence

**Figure 4:**
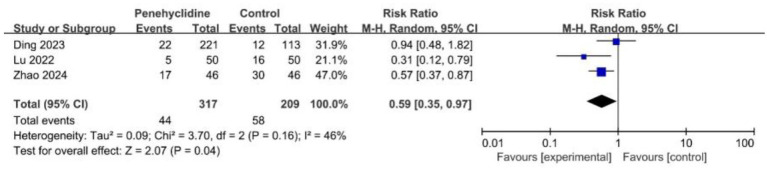
Forest plot of the incidence of PON between penehyclidine and control groups. PON, postoperative nausea.

Three trials assessed the incidence of PON. The forest plot revealed a significantly lower incidence in the PHC group (RR = 0.59, 95% CI [0.35, 0.97], *p* < 0.05, *I*^2^ = 46%, Figure 4), indicating low heterogeneity among the studies.

##### POV occurrence

**Figure 5:**
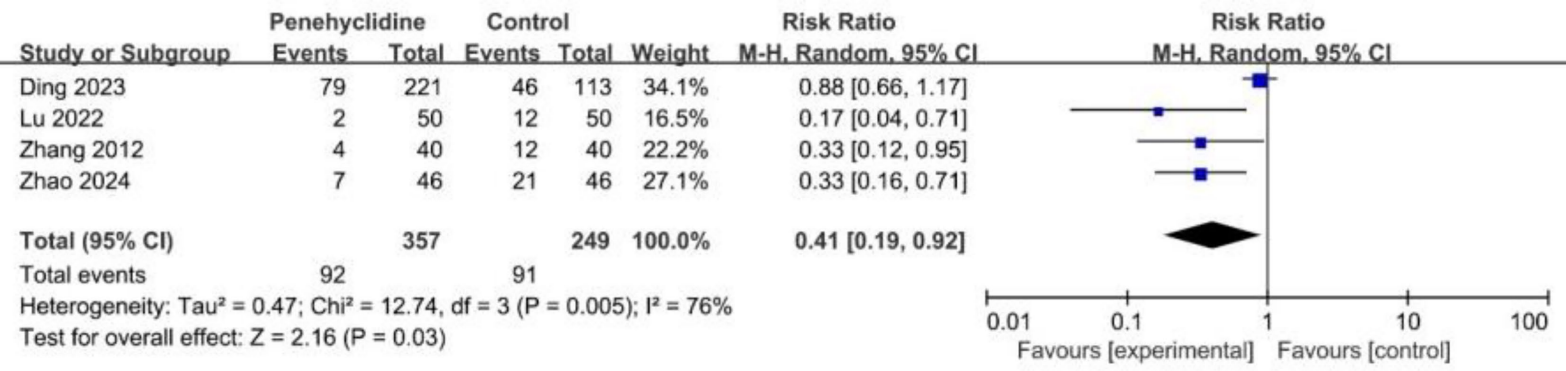
Forest plot of the incidence of POV between penehyclidine and control groups. POV, postoperative vomiting.

Four trials examined the incidence of POV. The forest plot demonstrated a significantly reduced incidence in the PHC group (RR = 0.41, 95% CI [0.19, 0.92], p < 0.05, *I*^2^ = 76%, Figure 5), reflecting high heterogeneity among the studies. Similarly, after excluding Ding et al.’s study, we re-conducted the meta-analysis, the result remained consistent, with reduced heterogeneity (Supplementary Figure 5).

##### Severe PONV occurrence

Four trials reported on the incidence of severe PONV, the forest plot analysis revealed a consistent direction of effect and a significantly lower incidence in the PHC group (RR = 0.50, 95% CI [0.34, 0.74], *p* < 0.05, *I*^2^ = 13%; Supplementary Figure 6). The low statistical heterogeneity (*I*^2^ = 13%) suggests that the treatment effect of PHC may be robust across these different definitions of severity.

##### Rescue antiemetic occurrence

**Figure 6:**
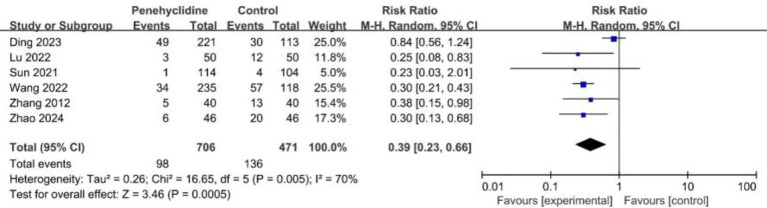
Forest plot of the incidence of required rescue antiemetic between penehyclidine and control groups.

Six trials evaluated the incidence of required rescue antiemetics. The forest plot analysis revealed a significantly lower incidence in the PHC group (RR = 0.39, 95% CI [0.23, 0.66], *p* < 0.05, *I*^2^ = 70%, Figure 6), indicating high heterogeneity. Similarly, after excluding Ding et al.’s study and redoing the meta-analysis, the result remained consistent, with reduced heterogeneity (Supplementary Figure 7).

##### Safety outcomes

**Figure 7:**
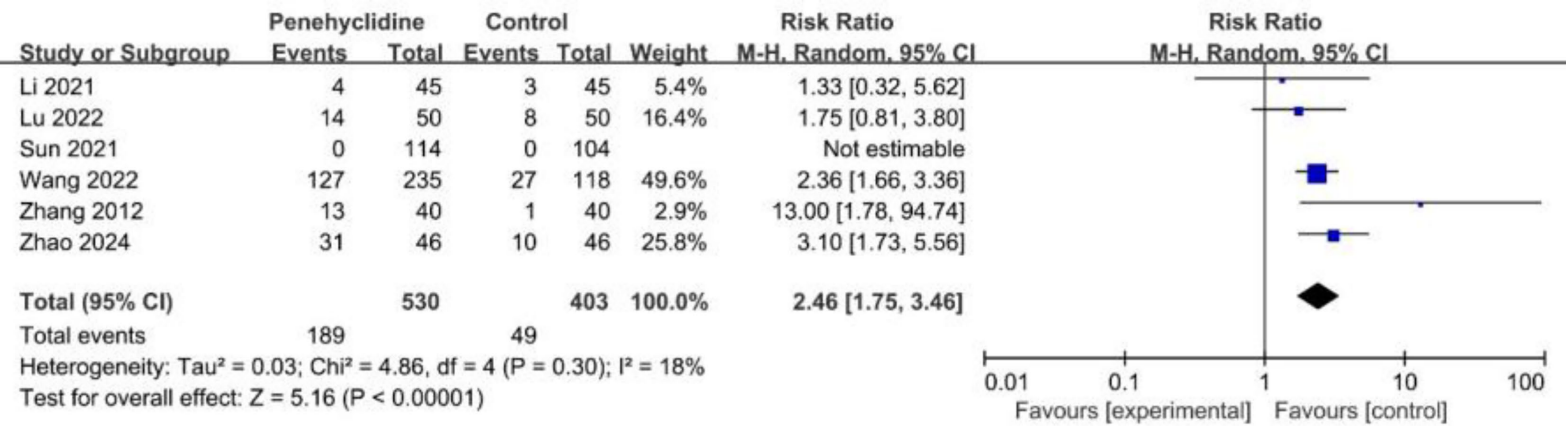
Forest plot of the incidence of dry mouth between penehyclidine and control groups.

Six trials evaluated the incidence of dry mouth. The forest plot analysis showed a significantly higher incidence in the PHC group (RR = 2.46, 95% CI [1.75, 3.46], *p* < 0.05, *I*^2^ = 18%, Figure 7), with low heterogeneity among the studies. Three trials reported the incidence of headache, and the forest plot analysis showed no significant difference between two groups (RR = 0.91, 95% CI [0.52, 1.60], *p* = 0.75, *I*^2^ = 0%, Supplementary Figure 8). Also, forest plot analyses showed no significant difference about the incidence of dizziness, urinary retention, fever between two groups (Supplementary Figures 9–11).

##### PACU stay

Three trials assessed the length of stay in the PACU. The forest plot analysis showed no significant difference between two groups (Supplementary Figure 12).

### Publication bias

A funnel plot was generated to visually assess potential publication bias (Supplementary Figure 13). While the plot appears largely asymmetrical, we note that the small number of included studies (*n* = 10) limits the interpretability of the plot (31).

### GRADE result

Table 2 shows the summary of the GRADE assessment.

**Table 2:** Summary for GRADE assessment.

Outcome	Included studies (*n*)	Patients (*n*)	RR/MD	95% CI	*I* ^2^	Quality of evidence	Reasons
Incidence of PONV	10	1,427	0.48	(0.36, 0.65)	68%	⨁⨁◯◯ LOW	“Imprecision” and “Other considerations” were downgraded to “serious.”
Incidence of PON	3	526	0.59	(0.35, 0.97)	46%	⨁⨁⨁◯ MODERATE	“Other considerations” was downgraded to “serious.”
Incidence of POV	4	606	0.41	(0.19, 0.92)	76%	⨁⨁◯◯ LOW	“Imprecision” and “Other considerations” was downgraded to “serious.”
Incidence of severe PONV	4	1,005	0.50	(0.34, 0.74)	13%	⨁⨁⨁◯ MODERATE	“Other considerations” was downgraded to “serious.”
Incidence of rescue antiemetic	6	1,177	0.39	(0.23, 0.66)	70%	⨁⨁◯◯ LOW	“Imprecision” and “Other considerations” were downgraded to “serious.”
Incidence of dry mouth	6	933	2.46	(1.75, 3.46)	18%	⨁⨁⨁◯ MODERATE	“Other considerations” was downgraded to “serious.”
Incidence of headache	3	272	0.91	(0.52, 1.60)	0%	⨁⨁⨁◯ MODERATE	“Other considerations” was downgraded to “serious.”
Incidence of dizziness	4	635	1.10	(0.72, 1.67)	0%	⨁⨁⨁◯ MODERATE	“Other considerations” was downgraded to “serious.”
Incidence of urinary retention	2	445	0.79	(0.25, 2.49)	0%	⨁⨁⨁◯ MODERATE	“Other considerations” was downgraded to “serious.”
Incidence of fever	2	445	0.90	(0.76, 1.07)	0%	⨁⨁⨁◯ MODERATE	“Other considerations” was downgraded to “serious.”
PACU stay	3	644	1.45	(−2.62, 5.51)	0%	⨁⨁◯◯ LOW	“Inconsistency” and “Other considerations” were downgraded to “serious.”

PONV, postoperative nausea and vomiting; PON, postoperative nausea; POV, postoperative vomiting; PACU, post-anesthesia care unit; RR, risk ratio; MD, mean difference; CI, confidence interval.

### TSA result

**Figure 8:**
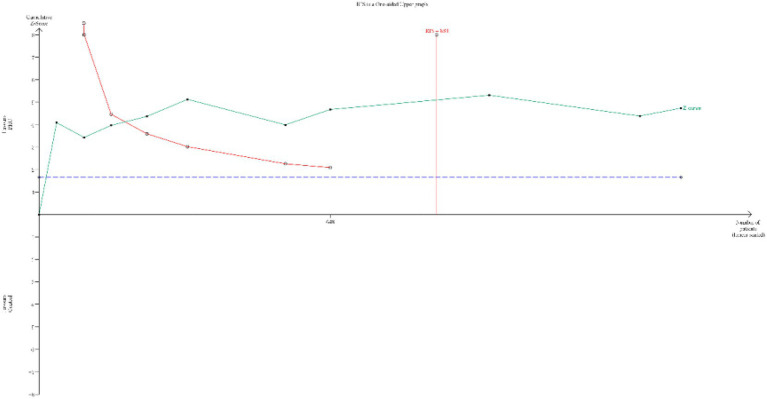
TSA for the incidence of PONV. (TSA, trial sequential analysis; PONV, postoperative nausea and vomiting) The cumulative Z-curve (green line) crossed the conventional test boundary (purple dotted line), TSA monitoring boundary (left red line) and required information size (right red line), which indicated that the evidence is sufficient.

The TSA for the primary outcome (incidence of PONV) is presented in Figure 8. The cumulative Z-curve crossed both the conventional significance boundary, the TSA monitoring boundary, and the RIS line. This indicates that the current evidence is might sufficient to conclude that PHC significantly reduces the incidence of PONV, and the risk of a false positive result is low.

## Discussion

To our knowledge, this meta-analysis is the first to examine both the safety and efficacy of PHC for preventing PONV after general anesthesia. Our results indicated PHC significantly reduced the incidence of PONV, as well as PON, POV, and severe PONV.

In our meta-analysis, we found that PHC significantly reduced the incidence of PONV, aligning with previous studies that explored PHC’s efficacy in reducing PONV in patients undergoing various types of surgery. For example, studies by Wang et al. (17) and Zhao et al. (26) also demonstrated that PHC effectively decreased PONV in patients undergoing gynecological and bariatric surgeries. Our pooled analysis of ten RCTs involving 1,427 participants provides a more robust evaluation of PHC’s effectiveness by integrating data from multiple settings and surgery types.

However, we observed differences in the results when comparing our findings to those of Ding et al. (19), who reported that PHC did not significantly reduce the incidence or severity of PONV in laparoscopic bariatric surgery patients. Also, this trial introduced significant heterogeneity into the results of this study. In the study, they enrolled patients who underwent laparoscopic bariatric surgery. The BMI was 38 (7) in both control group and PHC group. Several factors may account for this outcome. First, the trial included obese patients with high vagal tone, and the dosage of PHC administered may have been insufficient to effectively inhibit the activation of enteric vagus nerve afferent pathways (32). Second, the pharmacokinetics of PHC in obese patients may differ due to variations in drug distribution volume, which warrants further investigation (33). Third, during laparoscopic bariatric surgery, gastric denervation might occur, potentially diminishing the efficacy of PHC in alleviating gastrointestinal smooth muscle spasms.

The use of opioids and inhaled anesthetics during the perioperative period is strongly linked to a higher incidence of PONV (3). Opioid-induced PONV is typically dose-dependent (34, 35), and the use of opioids for postoperative analgesia prolongs the duration of PONV (36). Volatile anesthetics are a major cause of PONV in the early postoperative period (within 6 h), exhibiting a dose-dependent effect (37). Previous studies have suggested that various interventions can help reduce the incidence of PONV, including total intravenous anesthesia (21), opioid-free general anesthesia (38), and the combination of general anesthesia with regional anesthesia (39).

In our meta-analysis, the pooled RR for PONV with PHC prophylaxis was 0.48, indicating a statistically significant reduction. Also, the result of subgroup analysis indicated that PHC’s efficacy persisted. An interesting pharmacological interaction worth noting is that PHC, as an anticholinergic agent, might not only prevent PONV directly but also counteract the nausea-inducing effect of neostigmine, which is commonly used to reverse neuromuscular blockade. This potential dual mechanism could partly explain its efficacy, but our analysis could not definitively separate this effect due to insufficient reporting of neostigmine use. Currently, 5-HT_3_ receptor antagonists are the most frequently used medications for preventing PONV (8). However, the risk of QT interval prolongation linked to 5-HT_3_ receptor antagonists is gaining heightened scrutiny (40). PHC is commonly administered via intravenous injection or intramuscular injection. It has a half-life of approximately 10 h and is typically given as a single perioperative dose. Currently, there is no available oral formulation of PHC (15). The prolonged action of PHC permits its once-only administration during surgery, which may help reduce the workload of nursing staff and simplify perioperative antiemetic protocols. In contrast, 5-HT₃ antagonists, particularly newer agents like palonosetron, tend to be more costly. Furthermore, the need for repeated dose or additional rescue antiemetics may increase the overall cost burden of traditional treatments. Limited pharmacoeconomic evaluations suggest that PHC may offer a favorable cost profile, especially in resource-limited settings. However, further direct cost-comparison studies are warranted to validate these findings.

The pathophysiological mechanism of PONV is closely related to muscarinic receptors (41). The vestibular system has many M1 receptors, and anticholinergics inhibit cholinergic transmission between the vestibular nuclei and the central nervous system, as well as between the medullary reticular formation and the vomiting center (42). Studies have indicated that M3 and M5 acetylcholine receptors may help reduce the risk of PONV by mitigating motion sickness (43). PHC is a new long-acting anticholinergic, exhibits both anti-muscarinic and anti-nicotine properties, providing robust central and peripheral anticholinergic effects (44). It shows strong selectivity for the muscarinic M1 and M3 subtypes of acetylcholine receptors (15). Given its pharmacological profile, PHC has been increasingly investigated for PONV prevention, with prior studies demonstrating promising results (17, 21). However, strong comprehensive evidence is still lacking. Furthermore, PHC is relatively inexpensive and requires only single-dose administration, which may improve cost-effectiveness and compliance in clinical settings. Given these factors, we believe that the exploration of PHC as either an adjunct or alternative to existing PONV prevention strategies is clinically and scientifically justified.

Furthermore, our analysis provides a comprehensive overview of the safety profile of penehyclidine. The most commonly reported adverse event, dry mouth, was predictable based on its anticholinergic mechanism and was generally mild and self-limiting. When directly compared to standard antiemetics based on our study, PHC demonstrates comparable efficacy to first-line agents like ondansetron and dexamethasone in reducing PONV incidence. This favorable benefit–risk balance, where significant PONV reduction outweighs manageable side effects, supports a dual role for PHC within a multimodal PONV prophylaxis strategy. It can serve not only as a viable alternative for patients who are intolerant or have contraindications to conventional antiemetics but also as a potent additive component, potentially enhancing efficacy when combined with other agents through its different mechanism of action.

One notable limitation of this meta-analysis is that all included studies were conducted in China. This geographic concentration may limit the generalizability of our findings to other populations. Ethnic and genetic variability may influence the pharmacokinetics and pharmacodynamics of PHC. For instance, variations in cytochrome P450 enzyme expression or muscarinic receptor polymorphisms across different populations could potentially impact drug metabolism, efficacy, and safety profiles (45). Therefore, the TSA suggested the evidence may be sufficient, but the geographical concentration and methodological limitations of the included trials indicate that larger, more rigorous multi-center trials, particularly outside of China, would be beneficial to confirm these findings and enhance their generalizability.

It is important to note that PHC is not currently approved by the FDA for the prevention of PONV. While our findings suggest that PHC may be a promising agent for the prevention of PONV, the current body of evidence remains limited in scope and geographic diversity. For countries where it is not yet approved, this study positions PHC as a promising candidate for broader clinical evaluation and formulary inclusion. Its proven efficacy and acceptable safety profile suggest that it could valuably expand the armamentarium against PONV, particularly for high-risk patients or in settings where existing options are limited or ineffective. Future head-to-head trials against established antiemetics and cost-effectiveness analyses would be invaluable to further solidify its global role.

This meta-analysis has several limitations that need to be addressed. First, despite including 1,427 participants, only ten eligible trials were analyzed, resulting in a relatively small sample size. Second, all included studies were conducted in China, which may limit the generalizability of the findings to populations of different racial or ethnic backgrounds. Third, insufficient data prevented us from performing subgroup analyses for various types of surgeries and patient characteristics. Fourth, our meta-analysis has analyzed only some adverse events; however, comprehensive data on other potential side effects (such as blurred vision and heart rate changes) were not consistently reported.

## Conclusion

In conclusion, the findings of this meta-analysis suggest that PHC may have potential in reducing the incidence of PONV. There is a clear need for further high-quality, multicenter RCTs, ideally conducted across diverse patient populations and in alignment with international regulatory standards. Such studies are essential to confirm the efficacy and safety of PHC for PONV prevention and to determine its potential role in routine clinical practice.

## Data Availability

The original contributions presented in the study are included in the article/Supplementary material, further inquiries can be directed to the corresponding author.
