# Supplementary material for: Penehyclidine in prevention of postoperative nausea and vomiting: a systematic review and meta-analysis of randomized controlled trials
Source: Front Med (Lausanne). 2025 Sep 30;12:1676087. doi: 10.3389/fmed.2025.1676087 (PMC12518294; doi:10.3389/fmed.2025.1676087)

Search strategies for each database

Pubmed

("penehyclidine"[Supplementary Concept] OR "penehyclidine"[All Fields] OR "penehyclidine hydrochloride raceme"[All Fields] OR ("penehyclidine"[Supplementary Concept] OR "penehyclidine"[All Fields])) AND ("nausea"[MeSH Terms] OR "nausea"[All Fields] OR "nauseas"[All Fields] OR ("vomiter"[All Fields] OR "vomiters"[All Fields] OR "vomiting"[MeSH Terms] OR "vomiting"[All Fields] OR "vomit"[All Fields] OR "vomited"[All Fields] OR "vomits"[All Fields] OR "vomitings"[All Fields] OR "vomition"[All Fields] OR "vomitting"[All Fields]) OR ("postoperative nausea and vomiting"[MeSH Terms] OR ("postoperative"[All Fields] AND "nausea"[All Fields] AND "vomiting"[All Fields]) OR "postoperative nausea and vomiting"[All Fields] OR "ponv"[All Fields]) OR ("vomiting"[MeSH Terms] OR "vomiting"[All Fields] OR "emesis"[All Fields]) OR "emeses"[All Fields])

Web of science

#1 ((((AB=(Nausea)) OR AB=(Vomiting)) OR AB=(PONV)) OR AB=(Emesis)) OR AB=(Emeses)

#2 (AB=(penehyclidine hydrochloride raceme )) OR AB=(Penehyclidine)

#3 #1 AND #2

Embase

#1 penehyclidine OR (penehyclidine hydrochloride raceme)

#2 (Nausea)/br OR (Vomitin) OR (penehyclidine hydrochloride raceme) OR (Emesis) OR (Emeses)

# 3 #1 AND #2

Cochrane

#1 penehyclidine OR (penehyclidine hydrochloride raceme)

#2 (Nausea)/br OR (Vomitin) OR (penehyclidine hydrochloride raceme) OR (Emesis) OR (Emeses)

# 3 #1 AND #2

China Network Knowledge Infrastructure, and Wanfang Database

#1 长托宁 OR 戊乙奎醚

#2 术后恶心OR 术后呕吐 OR术后恶心呕吐

# 3 #1 AND #2

Supplement figure 1. Forest plot of the incidence of PONV between penehyclidine and control groups by excluding high heterogeneity study. (PONV, postoperative nausea and vomiting)


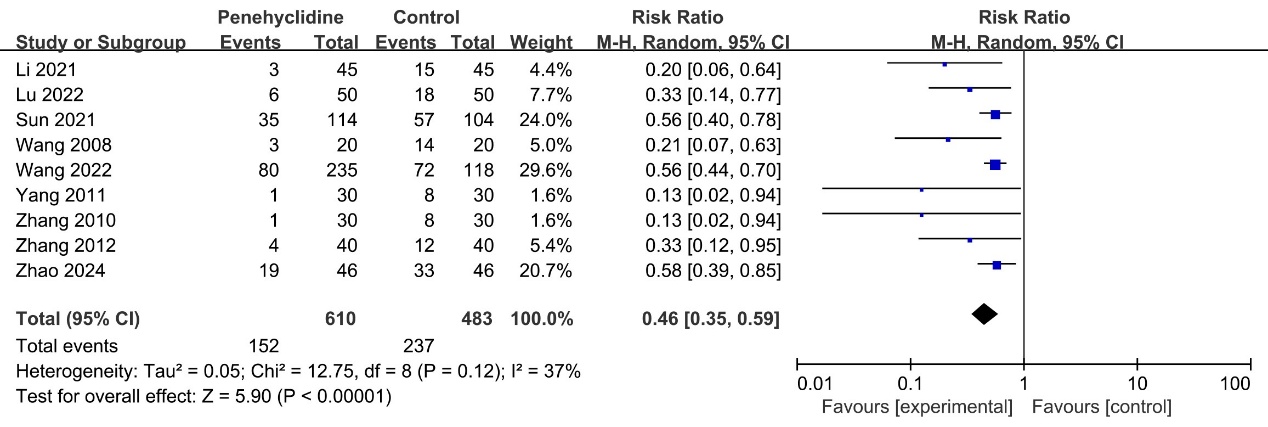


Supplement figure 2. Subgroup analysis for the incidence of PONV between penehyclidine and control groups according to different type of anesthesia. (PONV, postoperative nausea and vomiting)


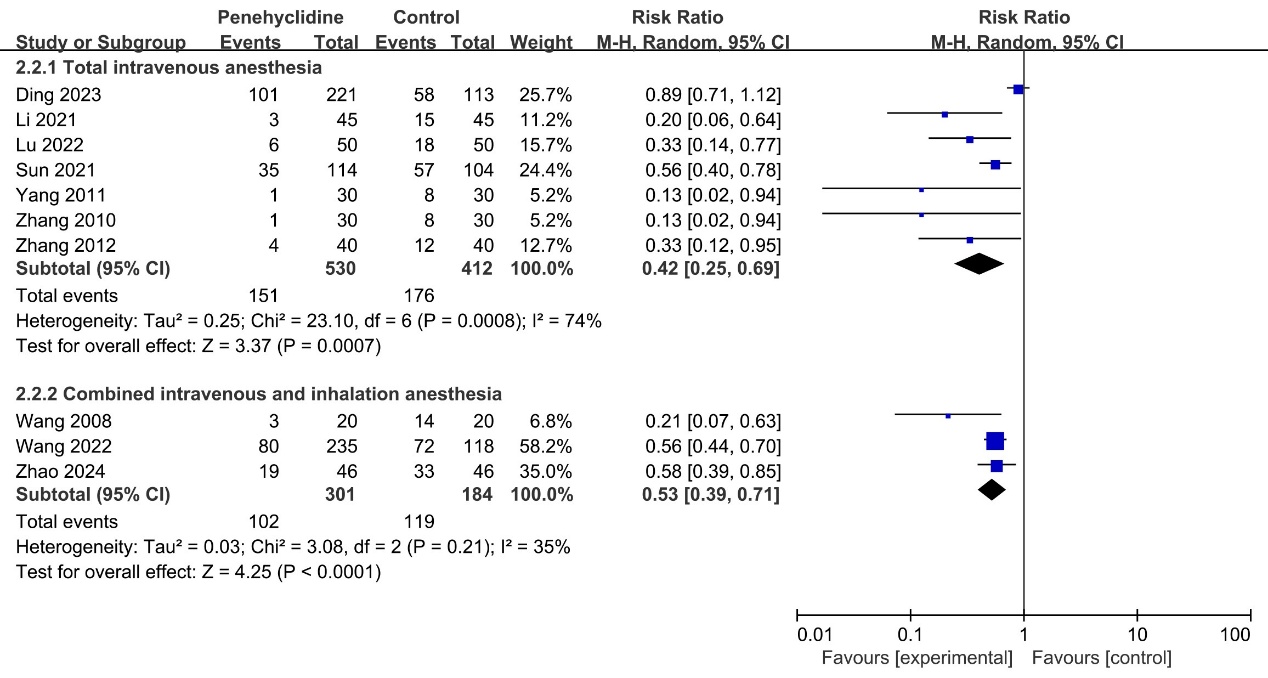


Supplement figure 3. Subgroup analysis for the incidence of PONV between penehyclidine and control groups according to different dosage of penehyclidine. (PONV, postoperative nausea and vomiting)


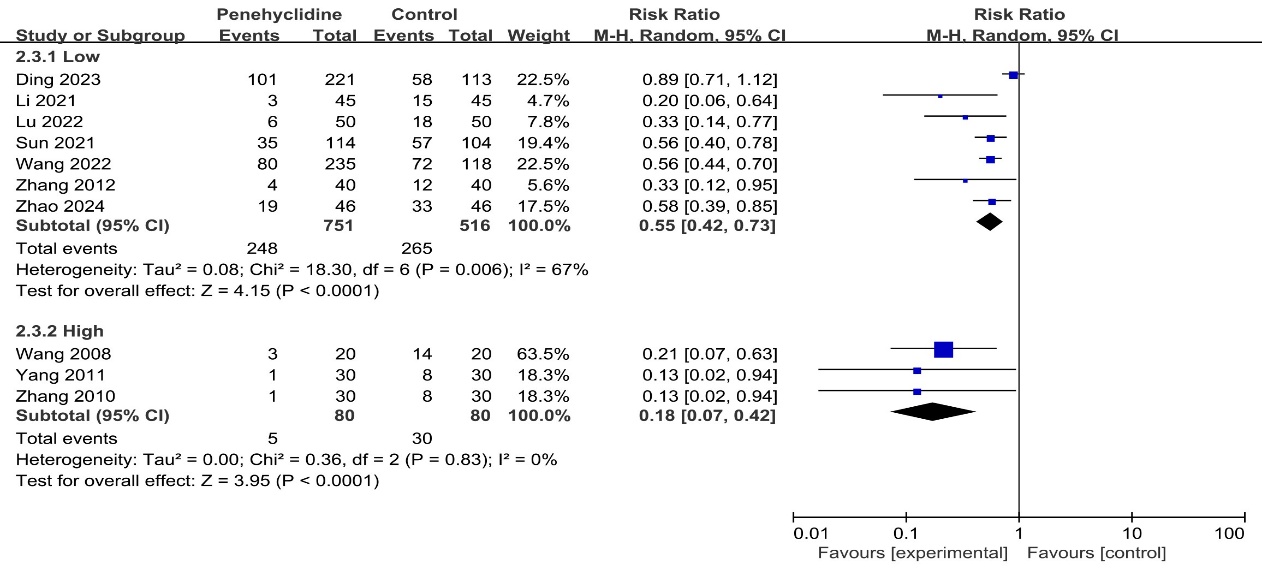


Supplement figure 4. Subgroup analysis for the incidence of PONV between penehyclidine and control groups according to different timing of penehyclidine administration. (PONV, postoperative nausea and vomiting)


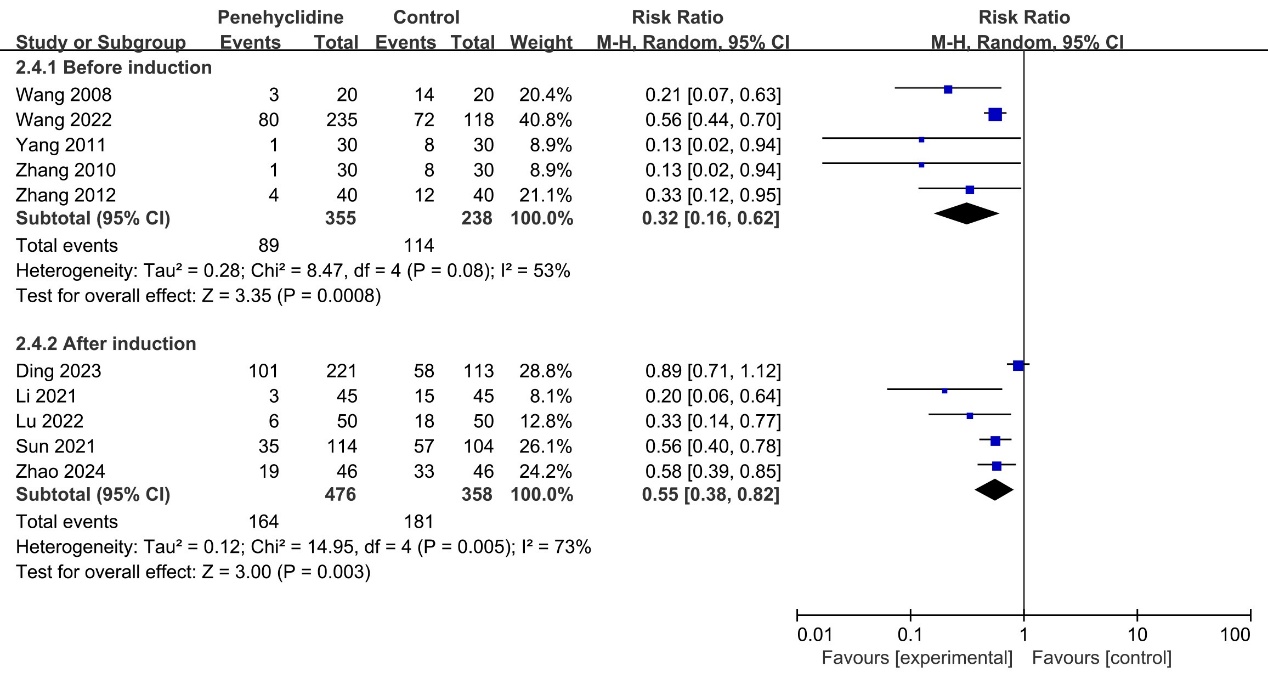


Supplement figure 5. Forest plot of the incidence of POV between penehyclidine and control groups by excluding high heterogeneity study. (POV, postoperative vomiting)


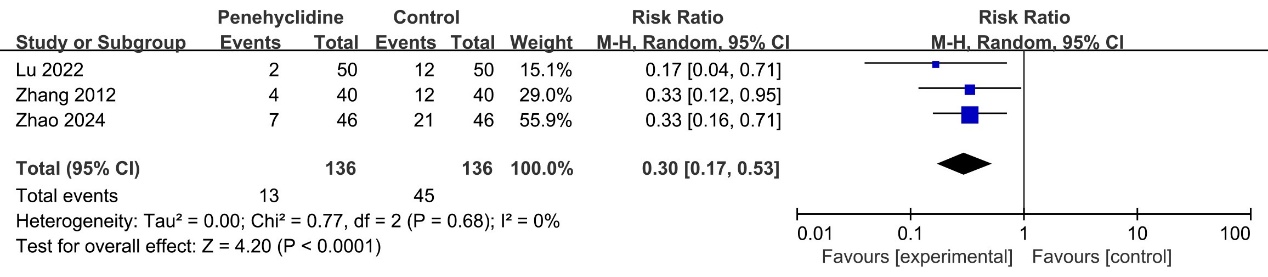


Supplement figure 6. Forest plot for the incidence of severe PONV between penehyclidine and control groups. (PONV, postoperative nausea and vomiting)


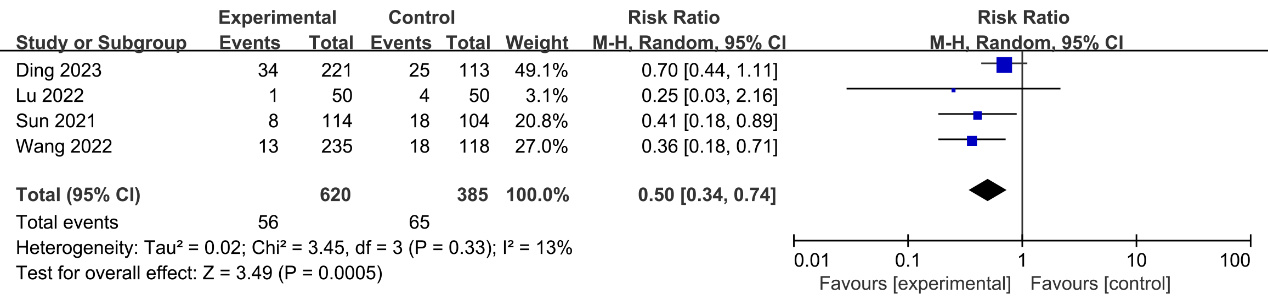


Supplement figure 7. Forest plot for the incidence of rescue antiemetic between penehyclidine and control groups by excluding high heterogeneity study.


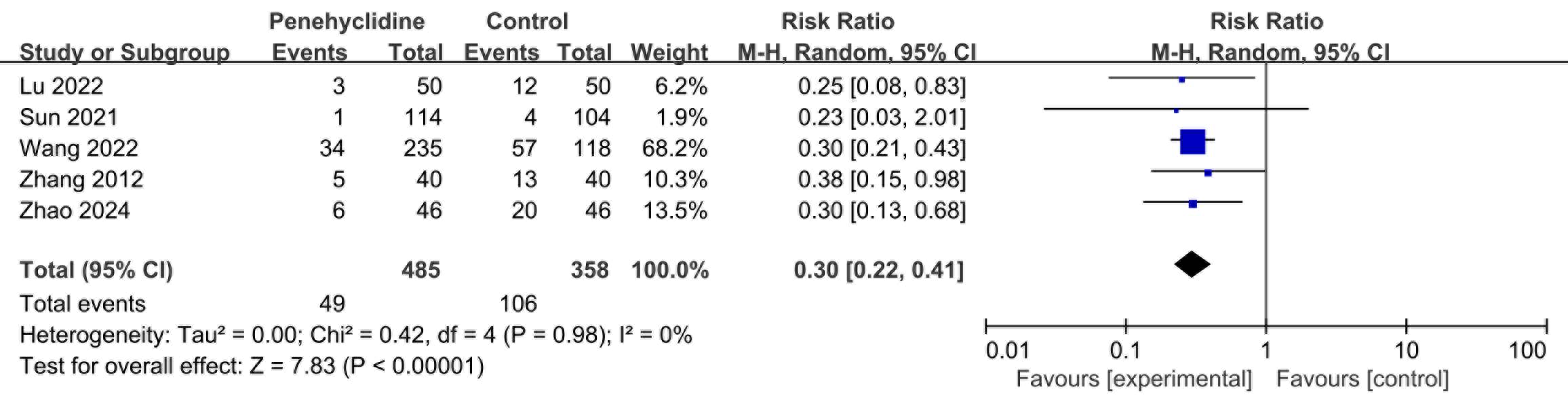


Supplement figure 8. Forest plot for the incidence of headache between penehyclidine and control groups.


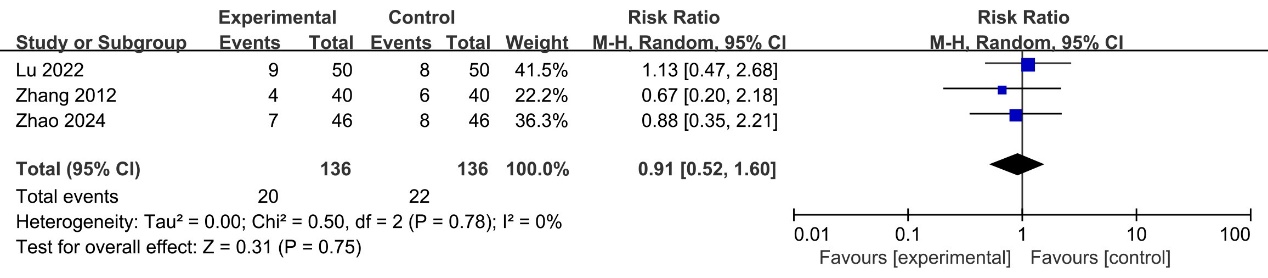


Supplement figure 9. Forest plot for the incidence of dizziness between penehyclidine and control groups.


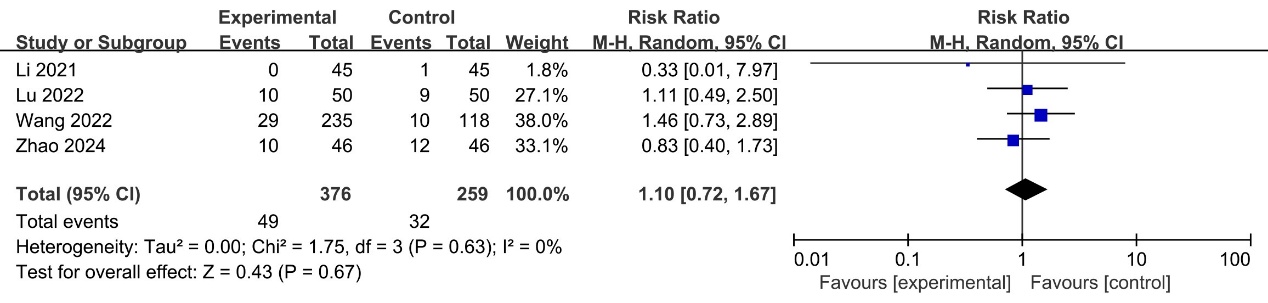


Supplement figure 10. Forest plot for the incidence of urinary retention between penehyclidine and control groups.


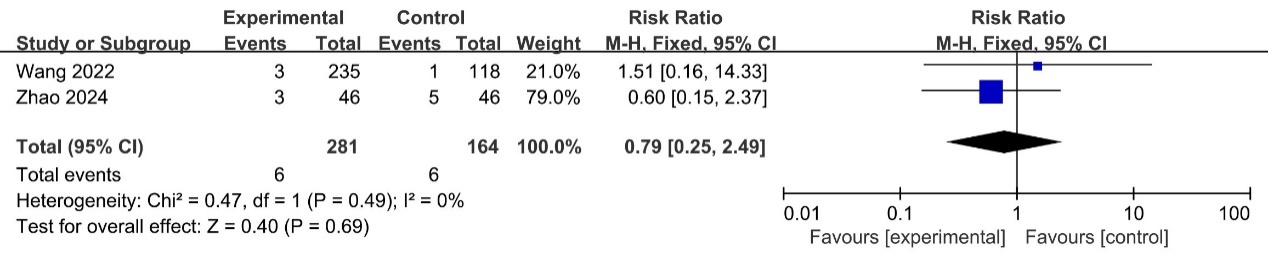


Supplement figure 11. Forest plot for the incidence of fever retention between penehyclidine and control groups.


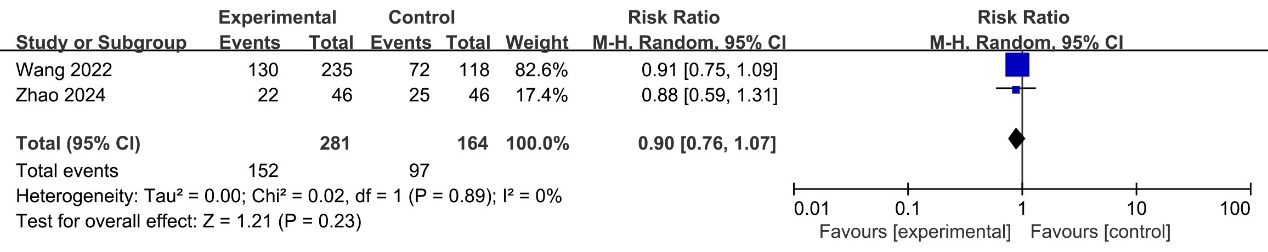


Supplement figure 12. Forest plot for the PACU stay between penehyclidine and control groups. (PACU, post-anesthesia care unit)


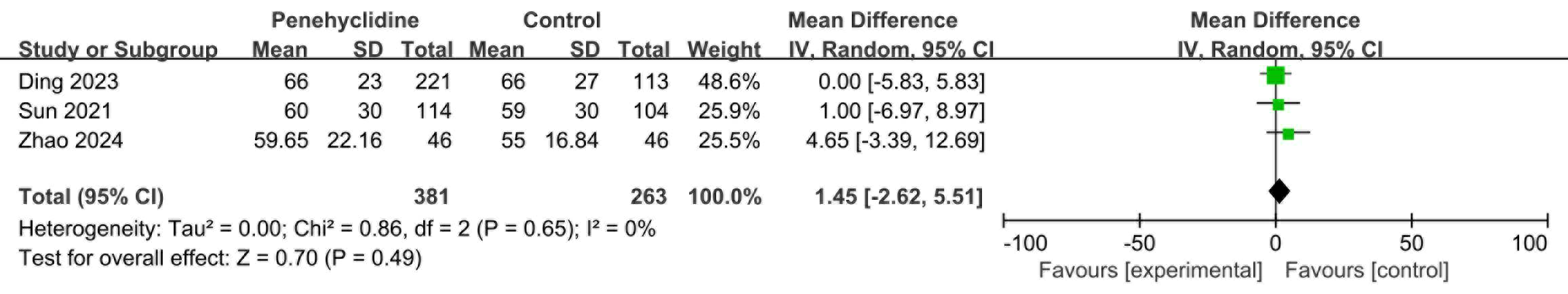


Supplement figure 13. Funnel plot for the incidence of PONV. (PONV, postoperative nausea and vomiting)


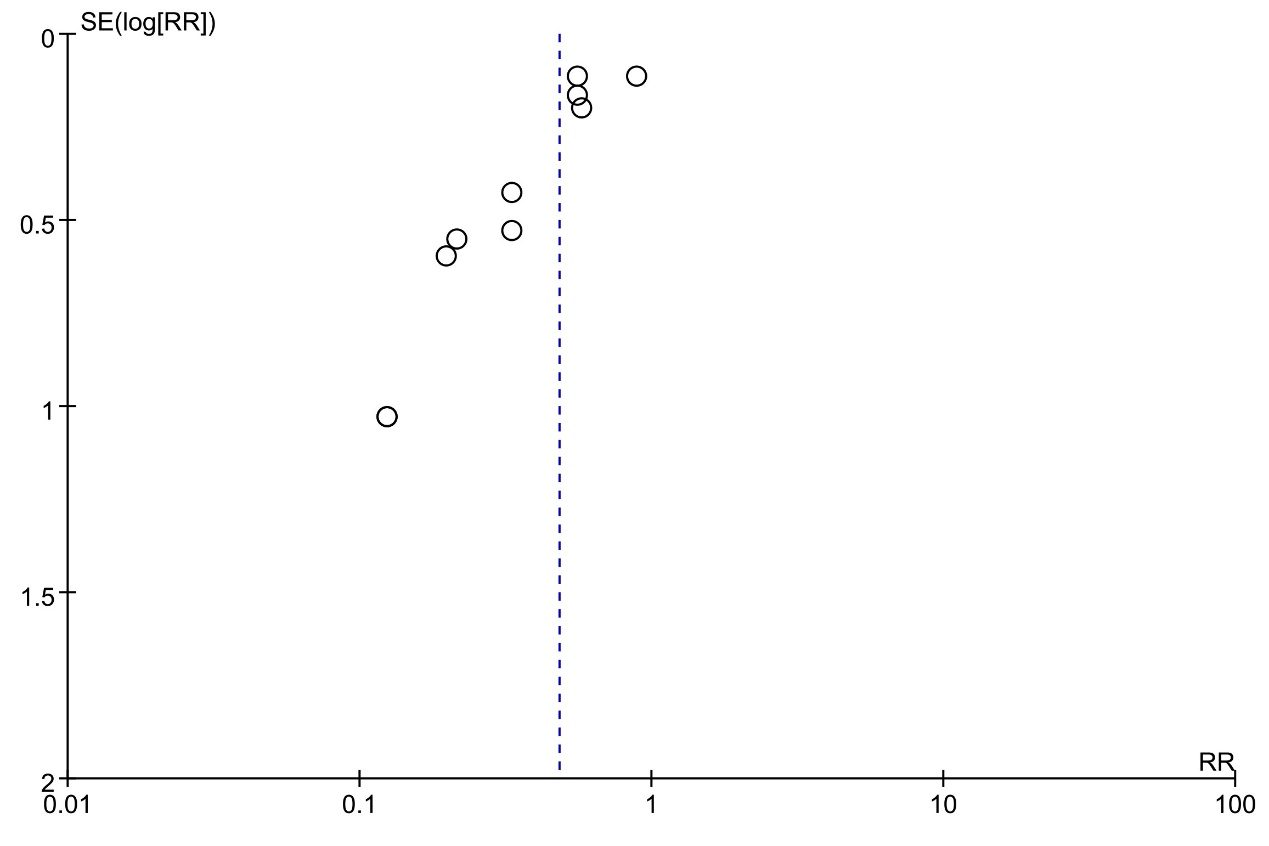

Supplement: Supplementary file 1 [file Supplementary_file_1.docx]
